# Exploring medical students’ early experiences of interacting with the multi-disciplinary team (MDT): A qualitative study

**DOI:** 10.15694/mep.2021.000030.1

**Published:** 2021-02-02

**Authors:** James Ainsworth

**Affiliations:** Swansea Bay University Health Board

**Keywords:** interprofessional education, IPE, education, medical education, multiprofessional

## Abstract

This article was migrated. The article was marked as recommended.

Background:

- Interprofessional education relates to educators and learners from two or more different health professions working together to create a collaborative learning environment which aims to improve patient care and teamwork. Part of a doctor’s role requires an understanding and respect for the multiple professions involved in patient care and an ability to work within an interprofessional team. Development of future NHS highlights a central role for IPE.
- This study aims to explore the early experiences that medical students have with the MDT, looking at IPE at Barts and London School of Medicine and Dentistry.
- It aims to understand students preconceptions of different professional roles involved in patient care, and the effect of these shadowing experiences on these views, and explore students opinions on what are the barriers to effective interprofessional collaboration.

Methods:

**Phase 1:** Thematic analysis of student reflections

-
  - 50 reflections used
  - Organised using Nvivo
  - Thematic analysis approach
  - Tree like framework of codes and subcodes

**Phase 2:** Focus group and interviews

-
  - More detailed exploration of key themes and issues
  - Total 50 minutes recording time
  - Same codes used and organised in NVivo

Results:

Students interacted with a variety of health professionals, developing awareness of interprofessional teamwork, and of the different roles involved in patient care. Students showed very limited prior knowledge and stereotyped views of some professional roles in healthcare, but demonstrated changes in attitude as a result of IPE, and were able to gain a greater understanding and appreciation of different professions.

Conclusions:

Students feel more comfortable approaching and learning from those who are actively involved in working and communicating with them and their team throughout their placement. It is important that opportunities for the students to shadow other professions are encouraged and supported as students find it challenging approaching and initiating these encounters themselves.

## Introduction

List of abbreviations

IPE: Interprofessional education

MDT: Mutidisciplinary team

GMC: General Medical Council

Barts: Barts and The London School of Medicine and Dentistry

CAIPE: Centre for Advancement of Interprofessional Education

### 1. What this study is about and why it was done

This study was carried out in order to explore the early experiences medical students have with the multidisciplinary team (MDT) on clinical placements during their MBBS programme.

Barts and the London School of Medicine and Dentistry (Barts) has an integrated style curriculum in which the students receive some clinical exposure from year 1 onwards, providing students with some early patient contact, and experience in a clinical environment. Second year students are required to shadow a non-doctor member of the MDT, and to reflect upon this experience. This was intended to be an opportunity for them to learn from a non-medical health professional, gaining a greater understanding of their role and of interprofessional collaboration. This study also highlights some of the barriers to students interacting with other members of the MDT, and suggests recommendations on what can be done to optimise their learning.

Interprofessional education (IPE) is a process through which students develop an understanding of the different roles involved in patient care and of the importance of effective teamwork and interprofessional communication. Part of a doctor’s professional role requires an understanding and respect for the multiple professions involved in patient care. Interprofessional collaboration is an essential aspect of a doctor’s role, and medical schools seek to prepare students for this (Barr, 2010).

### 2. The aims of this study were:

• To explore students experiences of interacting with the MDT
• To understand students preconceptions of different professional roles involved in patient care, and the effect of these shadowing experiences on these views
• To gain an understanding of students perceptions of teamwork in the clinical environment
• To explore students opinions on what are the barriers to effective interprofessional collaboration

### 3. Literature Review

In recent years there has been an increase in interest and the amount of research carried out in the field of IPE. Since the 1960’s, the necessity for promoting effective team functioning and the importance of collaboration amongst healthcare professionals in providing a high standard of patient care has become more apparent. IPE is seen a means of implementing this, resulting in improvements in patient safety and care, and reducing overall costs (Simin *et al*., 2010; Sierpina and Kretzer, 2014; Bridges *et al*., 2011; Brown *et al*., 2006). More than 750 articles relating to IPE have been published since 2011 (Pawlina and Drake, 2015). There is a growing awareness of the fragmentation of healthcare delivery, particularly in areas such as chronic disease, geriatric medicine, and mental health, and the need for improvements in interprofessional training and practice (Smits *et al*., 2014).

The driving forces behind the growing interest in IPE are: the changing healthcare system; increasing knowledge and specialities; complex problems and care pathways; and the gradual educational reform towards a more student led learning experience, away from that of the didactic lecturer (Klein, 2005). Health professionals are moulded to adopt a discipline based approach to their practice. Collaboration requires changes to the paradigm of the education system (D’Amour *et al*., 2010).

IPE is an important step in the advancement of health professional education and improving the quality of patient care, enabling future doctors and other healthcare professional to be able to work effectively together in an ever more complicated and specialised healthcare system. IPE is also a necessary educational component in breaking down the barriers that prevent effective teamwork and communication in the workplace (Humphris and Dean, 2004; Zwarenstein *et al*., 2013). The structure of the NHS is hierarchical in nature and many individuals are resistant to change. Early education can help reform and combat negative stereotyping and attitudes, increasing levels of respect and trust between different professions (Aston *et al*., 2012).

On commencing medical school, students have limited knowledge of the different roles in healthcare (Parsell and Bligh, 1998). Medical students also have preconceived views, considering doctors to be the most valuable members of the MDT, unaware of the extent to which other professions are vital in providing the best possible care for patients (Booysen *et al*., 2012; Price *et al*., 2016). Evidence shows that undergraduate medical school can negatively impact student attitudes and perceptions, increasing cynical attitudes and decreasing humanitarian feelings, due to a biomedical emphasis and little on the broader range of attitudes and social issues in medicine (Price *et al*., 2016; Pitout *et al*., 2016; Wolf *et al*., 1989). Early experience may better facilitate later interprofessional collaboration in practice, and foster favourable perceptions of IPE (Pawlina and Drake, 2015; Ewan, 1988; Lerner, Magrane and Friedman, 2009; Kitts, Christodoulou and Goldman, 2011; Harden, 2015).

In 1987 an organisation called CAIPE, or the Centre for Advancement of Interprofessional Education, was established, committed to the promotion and development of IPE in the UK. They define IPE as “educators and learners from 2 or more health professions and their foundational disciplines who jointly create and foster a collaborative learning environment. The goal of these efforts is to develop knowledge, skills and attitudes that result in interprofessional team behaviours and competence” (Mellor, Cottrell and Moran, 2013; CAIPE, 2016). Students must be exposed to an environment where multiple different professions are working together to improve health outcomes in order for IPE to be effective (Milton, 2012; WHO, 2010; Teodorczuk *et al*., 2016; Thislethwaite, 2010).

The benefits of improving partnership in healthcare result in improved patient care and outcomes and cost saving. There are also benefits for those working within the NHS; better working environment, greater staff satisfaction, improved communication and organisation, and reduced conflict (Turrentine *et al*., 2016; Mirjam, 2010).

Students need to be educated on how to function within a team, addressing the different roles, communication skills, and conflict resolution (Barr, 2015). In order to prepare students in becoming the next generation of doctors, students need to be introduced to a wide range of health professionals during their education, and universities must incorporate interdisciplinary experiences into their curriculum (Hall and Weaver, 2001). Different healthcare students working together during their education and training, the concept of ‘shared learning’, is an effective way of fostering a greater understanding and respect of different roles, and improving interprofessional communication and teamwork (Lavin *et al*., 2001; Pitout *et al*., 2016).

There are organisational barriers to IPE at many levels, such as rigid curriculums, lack of perceived value and lack of time and resources. In order for IPE to be effective more time and resources must be given to its development, and requires the commitment of those involved. It is vital that the importance of IPE is acknowledged and that mandatory sessions are included in the curriculum, as students interpretations of interprofessional collaboration are often largely shaped by the ‘hidden curriculum (Horsburgh, Lamdin and Williamson, 2001; Burning *et al*., 2009; Hays, 2013). There is an increasingly large amount of support and evidence behind IPE, but it is still not the norm in most universities and progress is still needed (Gwee, Samarasekera and Chong, 2013; Wingo *et al*., 2015; Ho *et al*., 2008; Aston *et al*., 2012; Daly, 2004).

IPE needs input from multiple professions, thriving when there is a sense of community, respect and equity, where open and mutual support is demonstrated (Bridges *et al*., 2011; Turrentine *et al*., 2016; O’Connell and Pascoe, 2004). Universities and health services need to work together. In order to achieve the greatest effect IPE should be carried out in the classroom and clinical setting (Weller, Barrow and Gasquoine, 2011; D’Eon, 2009). Due to the ‘culture’ of the NHS IPE is not always effective, and a restructuring of the education system is necessary to reduce the hierarchical, uniprofessional structure (Mirjam, 2010; Martin *et al*., 2004).

## Methods

Each student was required to submit a reflection following an opportunity to shadow a non-doctor member of the MDT. On submission, students were given information about the project and notified that their reflections might be used for educational research. The data was to be anonymized and students were able to opt out.

The initial phase entailed looking at the content of student’s reflections to identify themes or issues that could be used as a coding index in order to analyse the data (Vaismoradi, Turunen and Bondas, 2013). Ten reflections were selected initially at random from a total of 263 submissions to familiarise with the content and create an initial coding framework. A further 40 reflections were then randomly selected to gather more information. A thematic analysis approach and NVivo software was used to organisethe reflections and the focus group and interview transcripts.

The next stage of the project was to explore key themes and issues raised in more depth through focus group/interview discussions. Invitation emails were sent out to all second year students via the student office, which included an explanation of the project and the purpose of the focus group/interviews, and a consent form which they were required to sign in order to participate. A total of 9 students responded. The 6 students who responded initially were selected to take part in a focus group. A further 3 were interviewed: one individual interview, and one joint interview. The purpose of the focus group was to gain a more comprehensive understanding of the students’ experiences, the benefits and hindrances to these learning opportunities, and how professional collaboration affects the learning and behaviour of students. A topic guide was created, and 8 questions (each with additional prompts) based on the coding index from analysis of the reflections. The questions for the focus group and interviews were based on the coding index created from the reflections, and the transcripts analysed using the same framework, again organised using NVivo. The focus group and interviews were recorded, amounting to approximately 50 minutes total recording time (30 minutes for the focus group, and 10 minutes per interview). These recordings were transcribed professionally and a smart verbatimtranscript requested. The 6 participants in the focus group were labelled Respondent 1- 6, the participant in the individual interview Respondent 7, and those in the joint interview Respondent 8 and 9.

## Results/Analysis

The data selected for the results summarisesthe process of organising these shadowing experiences with different members of the MDT, student’s experiences during the shadowing, and some of benefits gained by students as a result of this opportunity. The data also allows exploration of student’s perceptionsof teamwork in the workplace and interprofessional collaboration.

### 1. Role of person shadowed

The task was for students to shadow a non-doctor member of the team. Despite this, however, 20% of students in the reflections shadowed doctors. The following table (Table 1), shows the different professions shadowed. The greatest proportion of these students shadowed nurses (42%).

**Table 1.** Detailed description of some of the roles shadowed in the reflections

Profession shadowed:	Details:
AHP	12 students (dietician, family therapist, speech and language therapist X2, occupational therapist (OT) X2, clinical psychologist X2, physiotherapist X2, support worker X2)
Medicine	10 students (psychiatrist X2, perinatal psychiatrist, surgeon, doctor X2, consultant nephrologist, paediatrician, junior doctor X2)
Other	2 students (psychiatry team member, chaplain)

The 9 students that took part in the focus group/interviews shadowed the following: doctors (2), pharmacist (1), physiotherapist (1), psychologist (1), and nurses (4).

### 2. Setting of the shadowing

The shadowing experiences took place in a variety of settings. The greatest proportion (48%) were in a clinical setting, such as on the wards or in clinics. A smaller number of students were in a non-clinical setting, such as an informal discussion or interview with the person shadowed, or an MDT meeting.

Of the 9 students in the focus group and interviews, 2 shadowed team members in clinics, 3 in MDT meetings, 1 attended a home visit, 1 on the ward, and 2 had informal discussions or interviews with the person that they shadowed.

### 3. Prior knowledge

As part of the requirement when completing the reflections, students were asked about the depth of their knowledge of the role of the professional they shadowed, prior to the shadowing experience. Very few of the 50 students whose reflections were analysed (8%) had a detailed knowledge of their role prior to the shadowing, with the majority (54%) having little or no prior knowledge of the role of the person shadowed. The remaining 19 (38%) had some knowledge.

Only 3 students mentioned the training or qualifications of the person/role that they shadowed. One student commented on the training of a psychologist, stating that their role is similar to a psychiatrist but they are not medically trained; one student commented on the training of a speech and language therapist; and another had some knowledge of the training of nurses.

The majority of the students who participated in the focus group and interviews had no, or very little, knowledge of the role of the person that they shadowed, and some were completely unaware of the existence of their role.

“*..community paediatrics was not something I was aware existed.” (Respondent 7).*

### 4. Questions asked

One requirement of the experience was for the students to think of 3 questions that they would like to find out/ask the person they shadowed, and to write these down, with answers, as part of their submitted work. Table 2 indicates the types of questions noted in their reflections. 50% of the students wished to find out more about the challenges involved in the role of the person shadowed, with only 8% asking about the rewarding aspects. Fifty percent asked questions relating to their role within the MDT. The majority of students (86%) had asked about the daily routine of the person shadowed. Thirty to forty-four percent of students asked questions relating to patients, and about skills relating to the role of the profession shadowed. Relatively few students asked about the working environment, the importance of their role, or the necessary training/qualifications.

**Table 2.** Questions asked by students in the reflections

Questions asked relating to the following aspects of their role:	No. of students:
Daily routine	43
Challenges	25
Role within the MDT	25
Relating to patients	22
Skills related to their role	17
Importance/purpose of their role	6
Training/qualifications	5
Rewarding aspects	4

### 5. Process

Twenty eight out of the 50 students whose reflections were analysed had had the shadowing experience set up for them, mainly done via their tutor. Occasionally an opportunity for a student to shadow a non-doctor member of the team was set up by one of the doctors on the placement, or rarely directly by someone approaching the student. Some students self-initiated the experience, normally someone that they had had previous contact with during their placement. Very few students (only 2 out of the 50) asked to shadow someone specific of interest to them, and none in the focus group/interviews.

A small minority of students (4% of the 50 whose reflections were analysed, i.e. only 2 students) said that they were not comfortable approaching someone whom they did not know to ask to shadow them. Some students, despite the task being to shadow a non-medical member of the MDT, did shadow doctors or surgeons.

“*I was nervous in approaching somebody that I didn*’*t know, especially a consultant considering I have spent such little time in a hospital before.”* (Reflection 39).

From the information gathered from the students in the focus group and interviews it is clear that there was a distinct lack of preparation by students prior to the shadowing experience. Most students were unaware who they would be shadowing prior to arriving on placement that day, allowing little time for preparation.

### 6. Experience/activities during shadowing

Student activities included observation of clinical procedures and communication with patients by the person shadowed, as well as some opportunities to communicate with patients themselves, and practice some practical skills, such as taking blood, an ECG, and blood sugar. Only one student mentioned having the chance to communicate with other members of the MDT during the experience.

*“..this placement has given me a great opportunity to interact with other members of a healthcare team..”* (Reflection 20).

Some students had a discussion with the person that they shadowed which helped increase their understanding of their role, and some attended an MDT meeting.

### 7. Increased understanding or awareness

Students reported a range of changes in their understanding of the roles of the professional shadowed that can be organised into the following categories:

• Challenges in their role:

In the reflections 54% of students commented on the challenges involved in the profession that they shadowed, the majority of which were relating to patients, administration, or specifics to the role (e.g. patient compliance or complaints, hospital guidelines and cuts to services, workload, etc.). Some also mentioned difficulties or challenges in the working environment, and relating to teamwork and communication within the team, and how poor communication and organisation can cause problems, for example missing or incomplete notes or handovers.

“*Unfortunately, the most challenging part was working with the Doctors. Whilst she stated that they were all amazing people and excellent clinicians, sometimes her job was made harder by lack of information on the referral sheets.”* (Reflection 40).

• Necessary skills or communication:

The majority of students (76%) gained an increased knowledge or awareness of the necessary skills or communication in the role of the profession that they shadowed. These were mainly in terms of communication skills with patients and with other members of the MDT. The findings were similar amongst the students in the focus group and interviews.

“*I thus learnt that it is very important to use appropriate wording, such that your take home message is both honest but also positive/encouraging, and not detrimental/offensive.”* (Reflection 1).

• Role within the MDT:

#### Responsibilities:

Thirty six students of the 50 students commented on the role of the person that they shadowed within the MDT, many appreciating the importance of their role in patient care.

“*I am surprised just how extensive the role of the social care workers and how vital they are to contributing to patients care, and emphasizing the need for a holistic approach in healthcare to completely satisfy the patients*’ *needs.”* (Reflection 16).

#### Interface with other professionals:

Thirty four students referred to the interfaces witness or discussed with the person shadowed between their profession and other health professions.

“*The nurse regularly checks patients*’ *blood. She also works with pharmacists, double checking that the correct medicines are prescribed and storing them for the patient to collect.”* (Reflection 13).

Many students in the focus group and interviews commented on the role of the MDT and the number of different professionals involved in patient care, and how patients are discussed amongst different health professionals in order to decide upon the best course of action. Some students also commented on the interface between different professionals in the pathways of patient care, for example between primary and secondary care.

The focus group and interviews were able to highlight student’s perceived barriers of effective teamwork, some of which are listed below:

- Differences in opinion between health professionals

- Prejudices, stereotypes, and a lack of understanding of different professions

- Lack of structure and organisation

- Poor administration

- Poor communication and relay of information between different professionals

- Lack of leadership

- Disagreements between doctors and other roles

“*..I think there can often be quite a divide sometimes where the doctor is more keen for actual pharmaceutical intervention..”* (Respondent 1).

“*Of nurses, definitely, like [name] was just saying you have such a narrow view I think of they are just there to support the doctors, and advise on the medication and stuff like that but they just do so much more. They*’*re the people that if someone doesn*’*t show up to their appointments, they*’*ll call and ask why didn*’*t you come and encourage them. They*’*re the intermediaries and it*’*s almost like they*’*re the most important person to get the patient to engage with the services.”* (Respondent 1).

Some students alluded to the stereotypes or prejudices, and the hierarchical nature of the NHS contributing to insufficient interprofessional collaboration.

“*..stereotypically the doctor might feel like they are bit more important because they are making the decisions about their care and stuff, but that could be frustrating for the nurses and the other members of the team who are there all the time and they actually know the patient..”* (Respondent 7).

• Values and ethos

The data contains some references to the different values and ethos between professions, often between the role shadowed and that of a doctor. Students felt that some roles spent more time with patients, formed stronger relationships, offered more emotional support and had a more holistic view of patient care. One student mentioned some conflict between a doctor and nurse on the ward, indicating the power dynamics and hierarchical nature of the NHS.

“*It was intriguing [sic] to experience the nursing side of things rather than shadowing [sic] doctors. There was more involvement with the patients, talking about their general well being [sic] and concerns. Their roles in keeping patients comfortable and showed what an important role they play. Nurses had a more personal relationship with the patient from spending more time with them.”* (Reflection 24).

Some students in the focus group commented on the differences in opinion between doctors and other professions.

“*.. I think there*’*s always a difference in opinions especially when you*’*re sectioning a patient, like, whether it*’*s the right thing to do; whether it*’*s ethically the right thing to do; if it*’*s the right thing for the patient; the right thing for the public..”* (Respondent 1).

### 8. What did students gain from the experience

It is clear from the reflections that the majority of students (78%) increased their knowledge of the role of the person shadowed: the challenges; importance of their role in patient care; communication with patients; correspondence with other professionals; and some commenting on necessary training/qualifications. Some increased their medical knowledge: medical conditions; patient management; drug treatments; procedures; epidemiology.

The majority of students also made personal developments as a result of the experience. Twenty four students stated that they enjoyed the experience or that it increased their confidence, for example in speaking to patients or other health professionals. Thirteen students said that the experience gave them motivation: to carry out further exploration into available/linked services within the NHS; to practice their own communication or practical skills; to engage more with non-doctor staff; to gain further experience; to further their medical studies; to advance their own career.

“*It inspired me to investigate further about the different organisations that tackle addiction and the possible volunteering opportunities that I could take part in.”* (Reflection 26).

Six students said that this experience has helped them adapt to new environments, for example in adapting communication skills to different patients, or dealing with sensitive issues. Eleven students said that they changed their point of view about the profession they shadowed: the importance of their profession in patient care; underestimated work load; the extent to which they are involved in patient management; the usefulness of different skills (e.g. non-verbal communication); importance of taking a holistic approach to patient care; importance of every member within the team and maintaining good communication.

“*I found out that social workers meet the both the psychological and basic needs of the patients. They take a holistic approach when supporting patients which can include the social, spiritual and psychological needs of the patient.”* (Reflection 23).

The experience allowed many students to develop their own communication or practical skills, as many had the opportunity to practice various procedures or communicate with patients or other professionals.

“*I felt my interprofessional communication certainly improved during the short time I was at the day care unit.”* (Reflection 30).

Thirty five out of the 50 students developed an increased respect for the profession they shadowed and the contribution made by their profession to patient care.

*“I feel like the role of clinical nurses is very underrated. The quality of care I witnessed in the initial counselling session went above and beyond what any doctor could have accomplished due to the very real time constraints of being a consultant or junior doctor. SH was able to really spend time addressing the concerns of the patient.”* (Reflection 28).

The data highlighted the value of interprofessional collaboration and effective teamwork in the workplace. Students who self-initiated the shadowing commented that this was easy as there seemed to be a supportive and approachable atmosphere amongst the team. Some students mentioned how problems and confusion can occur if there isn’t effective communication between individuals at the different stages of patient care. Students felt much more aware following this experience that doctors cannot provide every aspect of patient care, and that there are multiple roles which are all essential in providing care to patients.

“*To help appreciate the importance of the MDT like we are sitting a medical degree but it*’*s obviously we are going to be working in such close contact with some of the other professions that it*’*s good to get like an initial ... it*’*s good to be able to have the time to shadow them and like other roles, whereas like if you were to never do that you would just qualify and be a doctor and then be working with them rather than fully understanding what they do, it might help you respect them as well.”* (Respondent 8).

## Discussion

### 1. Prior knowledge and student misconceptions

This study shows that the majority of second year students have very limited knowledge of the different professional roles involved in patient care, many completely unaware of the existence of some professions, having a slightly simplistic interpretation of healthcare and of the number of stages and people involved.

*“I think it surprised me how many people are involved in the care of someone who has HIV. I don’t know. I just always thought ‘doctor, pharmacist, nurse.’ Pretty much, that’s it but they have Social Care Coordinators, Social Health Advisors and just everyone to make sure that you’re okay every step of the way and they deal with all the different aspects that you might be worried about. I didn’t realise there was so much care.”* (Respondent 1).

Previous studies have also shown that students have more knowledge of some roles than others, and has also highlighted negative relationships between different roles, for example between general practice and social work (Barr, 2010). Studies show that students have negligible knowledge of some roles in particular, such as that of the dietician (Wolf *et al*., 1989), and lack full understanding of the scope of other professions, for example that physiotherapists also deal with respiratory problems (Lerner, Magrane and Friedman, 2009).

It is clear that there are still stereotypes amongst current students. Students have a different view of the responsibility of a doctor compared with other roles, the relationships they form with patients, and the care that they provide. For example some students in this study were under the impression that doctors do most work, providing the principle care for patients, with nurses and various other professions being there to support them. Some students perceived that the role of the doctor is primarily concerned with treating disease, with less emphasis on the overall care of the health and well-being of the patient, a view that in many cases is supported by their experiences in clinical practice.

### 2. Role of IPE

Some form of IPE is necessary to increase students understanding of the different roles within the NHS, and how they are able to work collaboratively. These opportunities are important in expanding students’ understanding of interprofessional care and the contribution made by non-doctor members of the MDT. Only with increased understanding and respect for the different professions can these negative stereotypes be broken down. Previous literature suggests that interprofessional education (IPE) is necessary in overcoming these prejudices and negative stereotypes (Humphris and Hean, 2004), and that this should occur early in medical education to prevent these negative attitudes being carried forward into the next generation of healthcare professionals, and to increase the levels of respect and trust between them (Aston *et al*., 2012).

Many students were aware of differences in the values and ethos between different professions during their placements. Students were however surprised by the extent to which the roles which they shadowed were involved with patients, and their importance in upholding the best possible care. Following the experience, students seem to be under the impression that a more holistic approach to care is taken by non-doctor members of the team, such as nurses and social workers. This data suggests that working with other professions, even for brief periods of time, can greatly increase the levels of respect that students have for different professions, and their appreciation of their role within the MDT.

Witnessing these alternative views may also allow students to alter their own approach to patient care. Working with other professionals in patient care can give students a greater appreciation of all aspects of patient care, and the need to attend to patient’s general and mental wellbeing as part of their own practice.

*“I have learnt that it is important to take a holistic approach when caring for patients. It is important to remember that the patients we care for are human beings who not only feel physical pain but have emotions, expectations and have relationships with many people. Therefore it is crucial to not only focus on the physical symptoms but also consider the emotional, social, spiritual wellbeing of the patient.”* (Reflection 23).

Some of the data from this study highlights the hierarchical nature of the NHS, and the negative impact this can have on interprofessional collaboration. Some students witnessed how differences in opinion can cause disagreements amongst the clinical team.

### 3. Difficulties and Improving IPE

The main benefits of this shadowing scheme were mainly that of gaining greater knowledge of one specific role, getting a snapshot of their daily practice, and were less related to the interaction between different professionals and teamwork in the clinical environment. Some of the literature suggests that simply exposing learners to one professional role is a weak strategy for IPE, and that students need to learn in an environment where two or more different professions are working together in order to incorporate the interactive element. This type of standalone education of a specific role has been called ‘multiprofessional’ learning as opposed to IPE. Students must also witness interaction between professions to achieve true IPE (WHO, 2010; Burning *et al*., 2009).

Some students felt that they did not get very much out of this experience as they were only observing, unable to take part in any activities or practice their own communication. Others felt uncomfortable talking to patients and health professionals that they did not know, and might have benefitted from some training beforehand.

Some additional preparation by the university would have been beneficial, as there was a clear lack of preparation and initiative taken by students, occasionally having a complete lack of awareness of the existence or at least the purpose of the scheme. Many felt this was partly due to poor organisation and promotion of the scheme by the university, many being unaware prior to arriving on placement who they would be shadowing or what they would be doing. Students felt that they would have benefitted from having done some preparation, but that this needed to be better facilitated by the university/placement organisers. The literature on IPE states that in order to be effective, it requires commitment from faculty and students, and adequate time and resources. IPE should be incorporated into the planned curriculum, maintaining an awareness of the effects of the hidden curriculum. Professional collaboration must be displayed by all professionals involved in student learning (Horsburgh, Lamdin and Williamson, 2001; Burning *et al*., 2009).

Several studies suggests that IPE should begin early in medical education, initially campus based, then progressing to in a clinical context, facilitating improved interprofessional collaboration in practice. Students require basic skills training, and need to be educated on how to function within a team. Interprofessional collaboration is not an automatic result of placing students amongst different people, but may need active training on working together and communication, and requires an understanding of roles and responsibilities (Barr, 2015). Some students in this study said that they would not feel comfortable approaching people who they did not know, and were often unsure of the role of many people on their placement, and that they would have benefitted from some education or introductions as to the different roles prior to starting placement, in addition to more training in conversing with other health professionals and patients.

This data suggests that students are much more comfortable approaching a person to shadow when it is someone that they have encountered previously or whose role they are familiar with. The importance of having a warm and welcoming team in a supportive environment makes students feel more comfortable and proactive. Several studies highlight the importance of positive attitudes amongst staff in fostering favourable perceptions of IPE amongst students (Pawlina and Drake, 2015; Horsburgh, Lamdin and Williamson, 2001). IPE thrives when open and mutual support is demonstrated in workplace, a sense of community and support being identified as key resources to IPE (Turrentine *et al*., 2016).

### 4. Limitations

Due to the small scale of this study, only looking at year 2 undergraduate MBBS students at Barts and the London, there is limited applicability of the findings to different settings, however the results are capable of adding to the current literature and increasing the data available on IPE. The results from this study apply to this given form of IPE, and may not be applicable to different types of IPE used in different settings, or amongst a different cohort.

Due to the small number of participants in the focus group and interviews (n=9), the results of this may not have been representative of the entire year 2 MBBS cohort at Barts, however the data in from the focus group and interviews is supported by the data in the reflections. Each interview was roughly 10 minutes, limiting the amount of data that could be collected.

The short time frame of this project (October to May) allowed little time for further follow up of these students to examine any lasting changes.

## Conclusion

Year 2 students at Barts and The London School of Medicine and Dentistry often have limited understanding and stereotyped views of different professions involved in patient care. These students need to learn from different professionals in order to gain an understanding and respect for their contribution to patient care, but also need involvement with multiple health professions within the MDT to develop their teamwork skills and a greater comprehension of interprofessional collaboration. It is important that these opportunities for students to shadow other professions are encouraged and supported as students find it challenging approaching and initiating these encounters themselves. Year 2 students at Barts feel more comfortable approaching and learning from those who are actively involved in working and communicating with them and their team throughout their placement. Universities should incorporate IPE into their curriculum, and it should be supported by all faculty involved. Doctors need to role model good interprofessional communication and collaboration within the workplace. Good interprofessional team-working enhances students learning.

## Take Home Messages

• IPE aims to improve the efficiency of teamworking and interprofessional collaboration in the workplace, improving the quality of patient care.
• Medical students often have a limited and stereotyped view of different professions involved in patient care.
• This study demonstrates changes in student attitudes and understanding as a result of IPE at Barts and The London School of Medicine and Dentistry.
• These learning experiences and IPE need to be incorporated into the curriculum as students often have difficulty initiating them themselves.
• IPE should involve multiple health professions and modelling of good interprofessional collaboration and teamwork.

## Notes On Contributors

**James Dafydd Ainsworth**: The researcher and article author. The author achieved a BSc in Medical Education in 2016, and MBBS in 2018, both from Barts and The London School of Medicine and Dentistry. Following completing his foundation training, he is ccurrently working as a clinical fellow in intensive care medicine. ORCID ID: https://orcid.org/0000-0001-5571-1983
